# Supplementary material for: Role of Strong Localized vs Weak Distributed Interactions in Disordered Protein Phase Separation
Source: J Phys Chem B. 2023 Apr 20;127(17):3829–38. doi: 10.1021/acs.jpcb.3c00830 (PMC10187732; doi:10.1021/acs.jpcb.3c00830)
Supplement: Supplementary file 1 — jp3c00830_si_001.pdf [file jp3c00830_si_001.pdf]

Supporting Information
Role of Strong Localized vs. Weak Distributed Interactions in Disordered Protein Phase Separation

Shiv Rekhi,[†] Dinesh Sundaravadivelu Devarajan,[†] Michael P. Howard,[‡] Young C. Kim,[¶] Arash Nikoubashman,[§] and Jeetain Mittal[*,†,‖,⊥]

†*Artie McFerrin Department of Chemical Engineering, Texas A&M University, College Station, TX 77843, United States*

‡*Department of Chemical Engineering, Auburn University, Auburn, AL 36849, United States*

¶*Center for Materials Physics and Technology, Naval Research Laboratory, Washington, DC 20375, United States*

§*Institute of Physics, Johannes Gutenberg University Mainz, Staudingerweg 7, 55128 Mainz, Germany*

‖*Department of Chemistry, Texas A&M University, College Station, TX 77843, United States*

⊥*Interdisciplinary Graduate Program in Genetics and Genomics, Texas A&M University, College Station, TX 77843, United States*

E-mail: jeetain@tamu.edu

Phone: (512) 699-4643

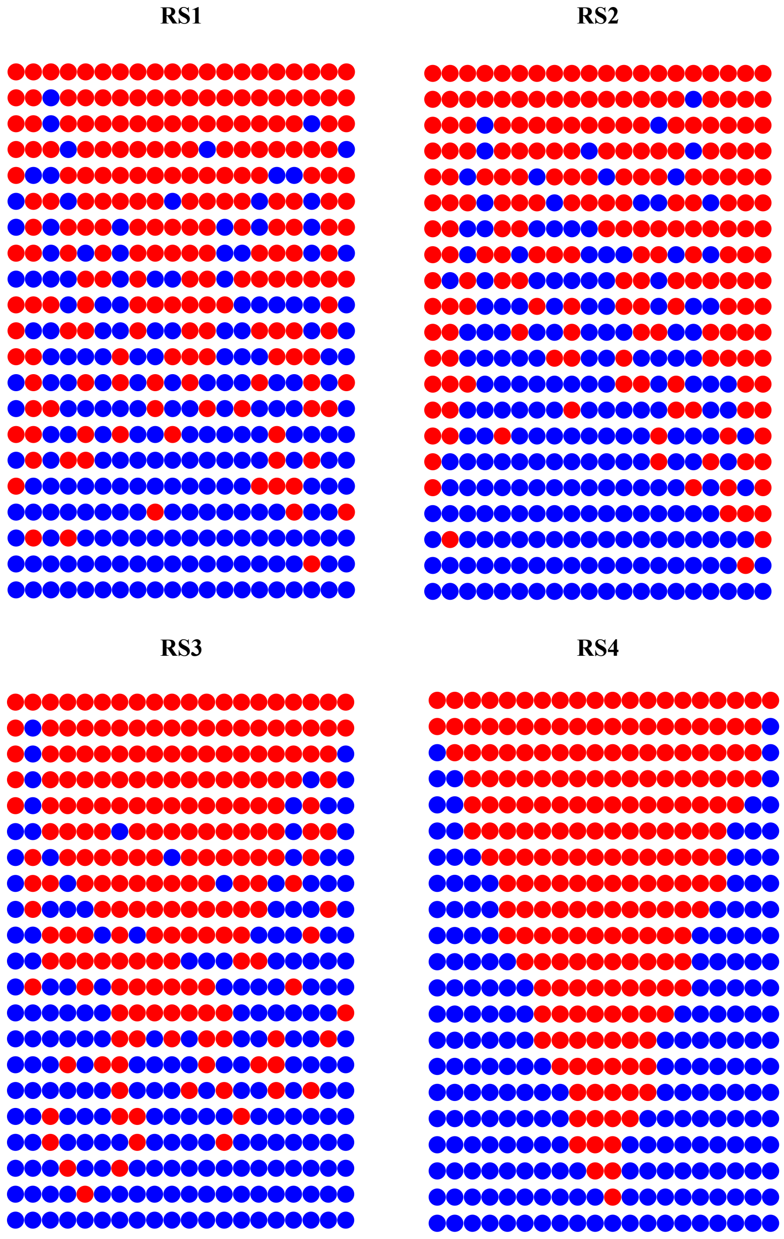

Figure S1: Three randomly generated sequence sets (RS1, RS2, and RS3) as well as a highly patterned sequence set (RS4). Red and blue beads correspond to hydrophobic (H) and polar (P) monomers, respectively.

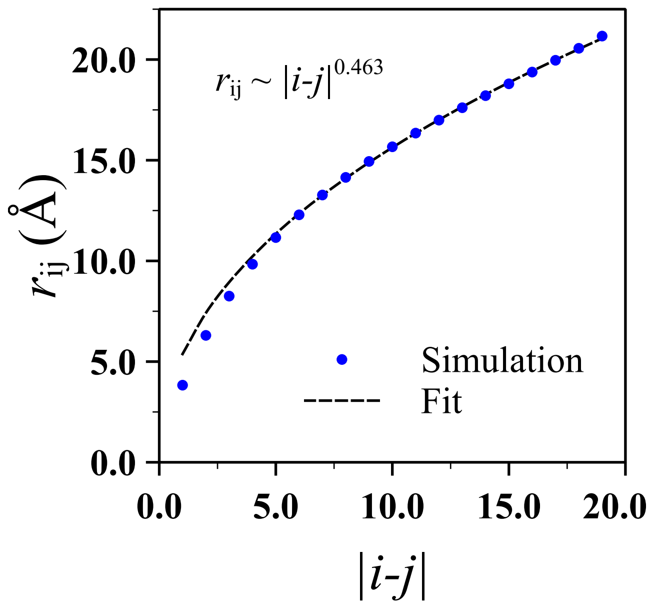

Figure S2: Intrachain distance r_{ij} as a function of sequence separation $|i-j|$ for the purely hydrophobic polymer $X_{\mathrm{P}} = 0$. Simulation data is shown as blue symbols while the fit to the data for $|i-j| > 5$ is shown as a dashed black line. The value of exponent is estimated to be 0.463.

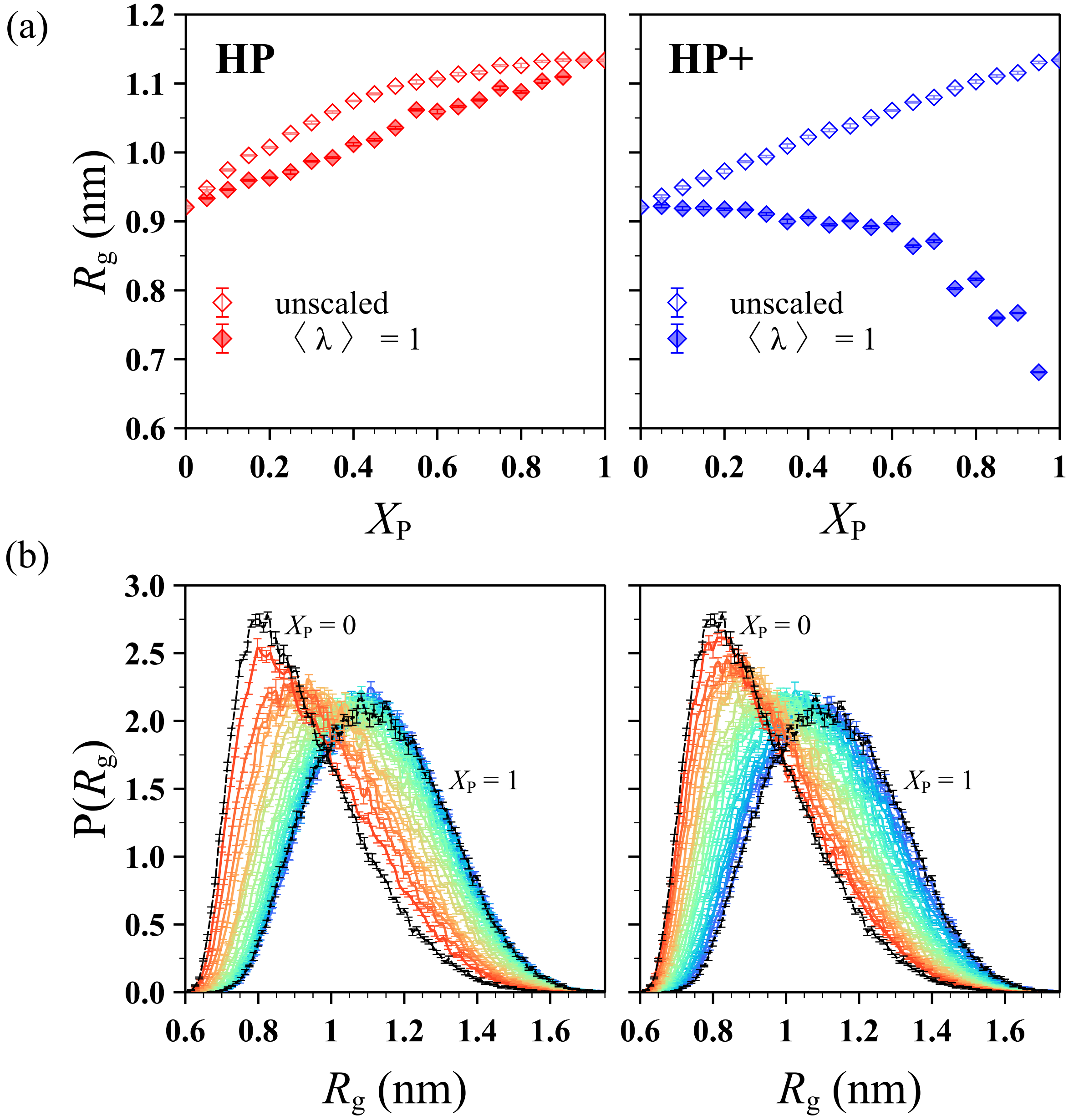

Figure S3: (a) Radius of gyration R_g as a function of X_P before scaling ($\lambda_H = 1$, open symbols) and after scaling ($\lambda_H = 1/(1 - X_P)$ that maintains average hydropathy per monomer $\langle\lambda\rangle$ of the chain at 1, closed symbols) for the HP (red) and HP+ (blue) models. (b) Probability distribution of R_g when $\lambda_H = 1$ for the HP (left) and HP+ (right) models. The line color, ranging from red to purple, indicates increasing X_P. The distributions for purely hydrophobic ($X_P = 0$) and purely hydrophilic ($X_P = 1$) chains are shown as dashed and dotted black lines, respectively.

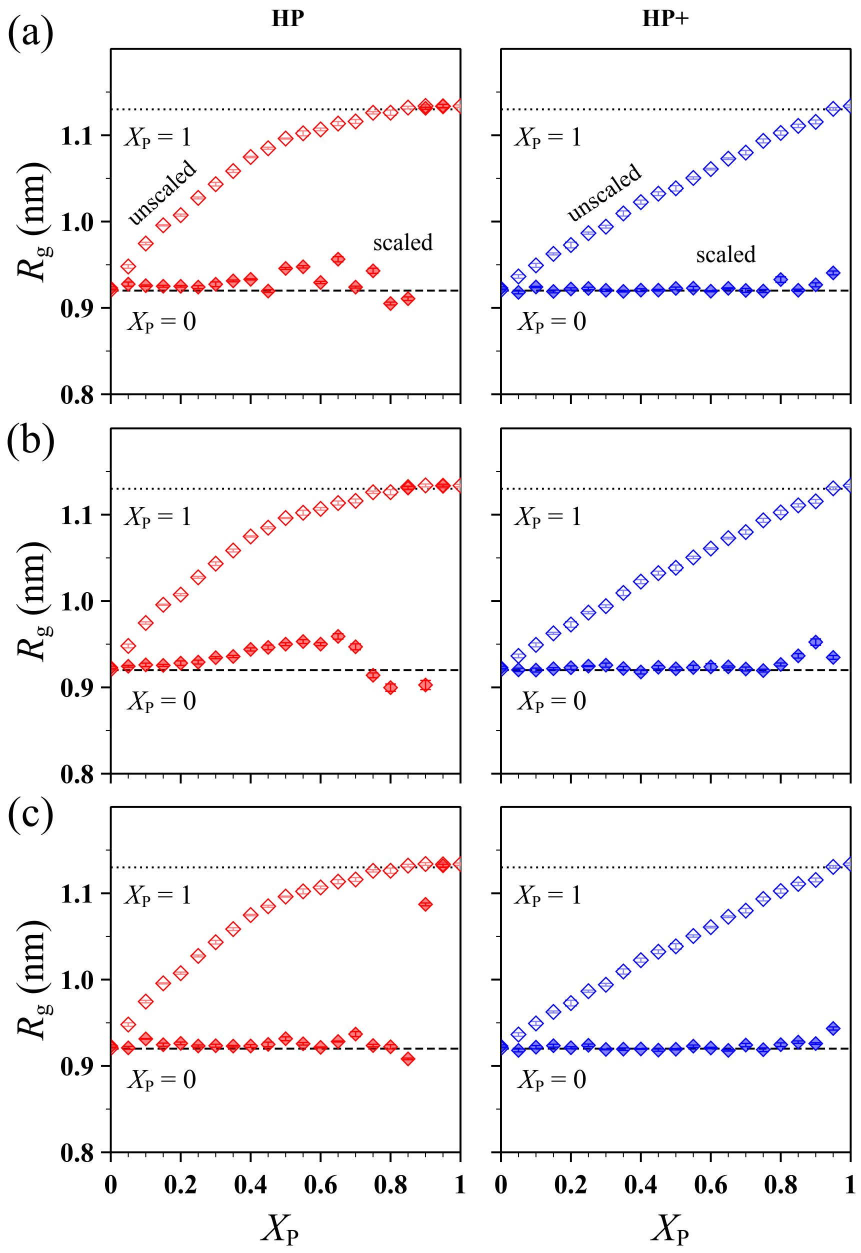

Figure S4: Radius of gyration R_g as a function of X_P before scaling ($\lambda_H = 1$, open symbols) and after scaling ($\lambda_H = a/(1 - X_P)$, closed symbols) for the HP (red) and HP+ (blue) models: (a) RS1, (b) RS2, and (c) RS3. Results for purely hydrophobic ($X_P = 0$) and purely hydrophilic ($X_P = 1$) chains are shown as dashed and dotted black lines, respectively, in all subplots.

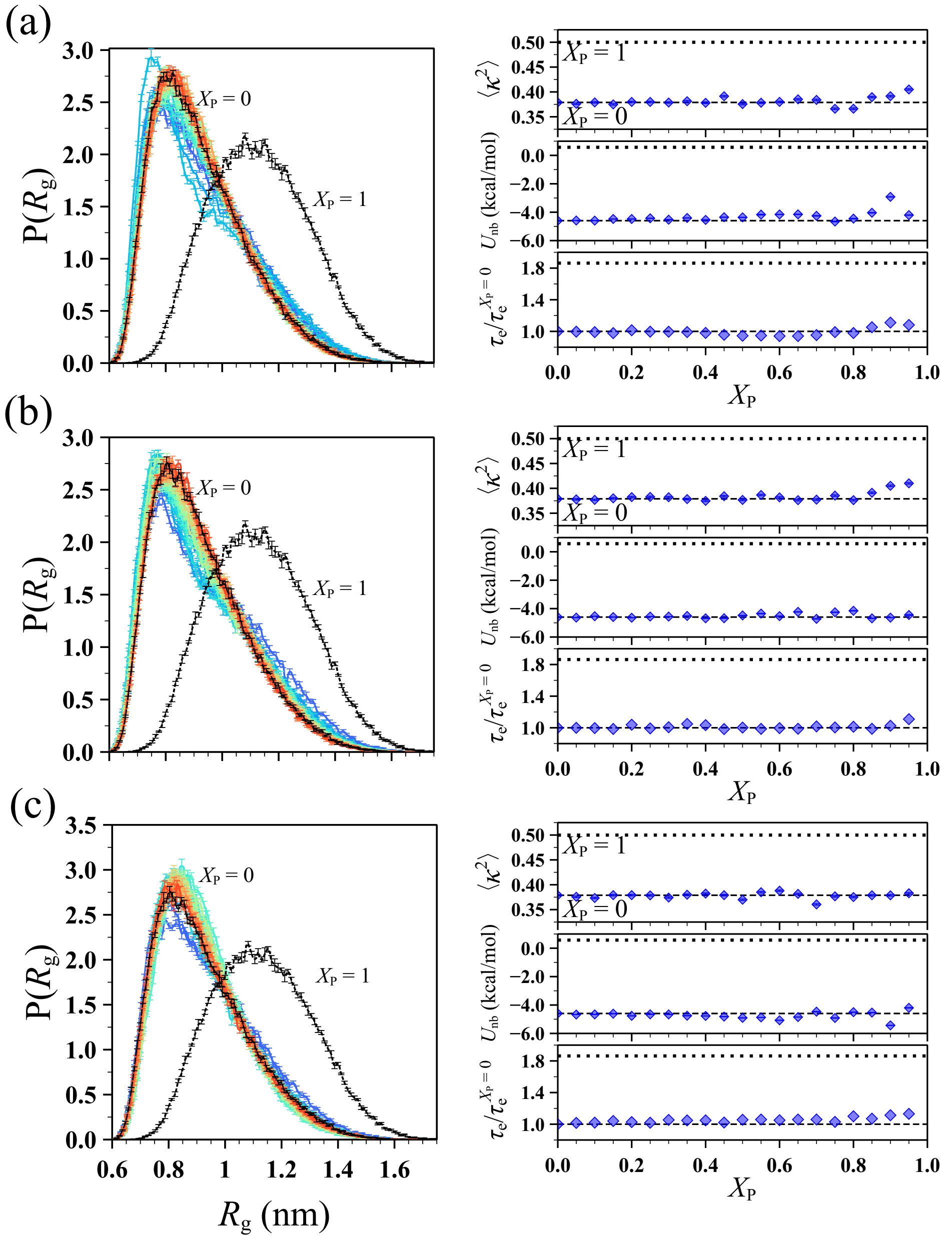

Figure S5: Probability distribution of radius of gyration R_g (left) and plots showing shape factor $\langle\kappa^2\rangle$, nonbonded potential energy U_{nb}, and end-to-end vector relaxation time τ_e as functions of X_P (right) for the HP+ model: (a) RS1, (b) RS2, and (c) RS3. In $P(R_g)$ plots, the line color, ranging from red to purple, indicates increasing X_P. The values of τ_e are normalized by that obtained for the purely hydrophobic chain ($X_P = 0$). Results for purely hydrophobic ($X_P = 0$) and purely hydrophilic ($X_P = 1$) chains are shown as dashed and dotted black lines, respectively, in all subplots.

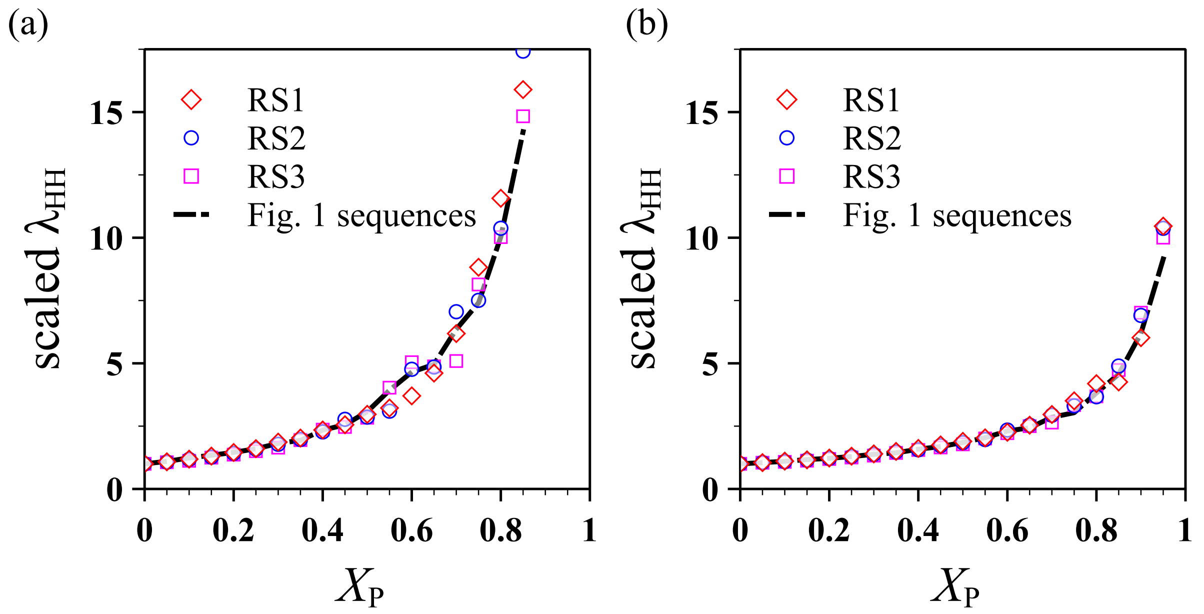

Figure S6: Comparison of scaled λ_{HH} [$\lambda_{\mathrm{HH}} = \lambda_{\mathrm{H}} = a/(1 - X_{\mathrm{P}})$] between the three randomly generated sequence sets (RS1, RS2, and RS3) as well as the primary sequence set shown in Fig. 1 for the (a) HP and (b) HP+ models.

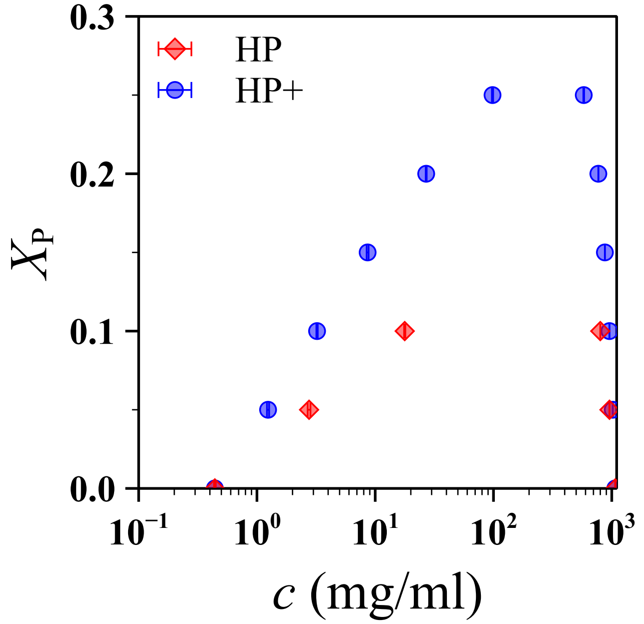

Figure S7: Phase diagrams for the HP and HP+ models when interactions are fixed at $\lambda_{\mathrm{H}} = 1$. Concentrations of dilute and dense phases for all X_{P} values were extracted from the corresponding concentration profiles.

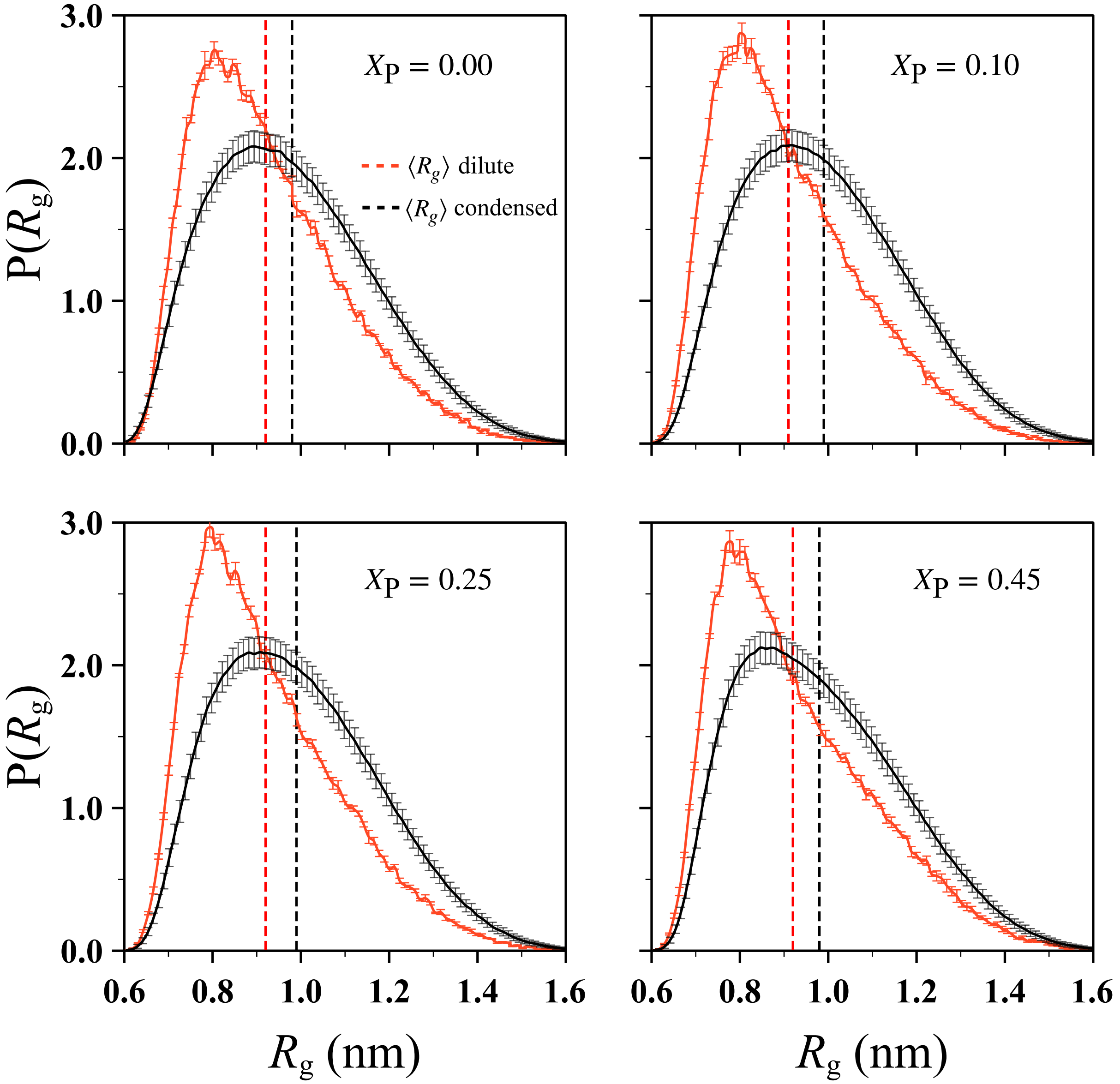

Figure S8: Probability distribution of radius of gyration R_g for the dilute phase (red) and condensed phase (black) for the HP model. The condensed phase values are computed as an average over all the chains in the system while the error bars are estimated as the standard deviation of R_g values among all chains. The dashed lines indicate the mean of the distributions.

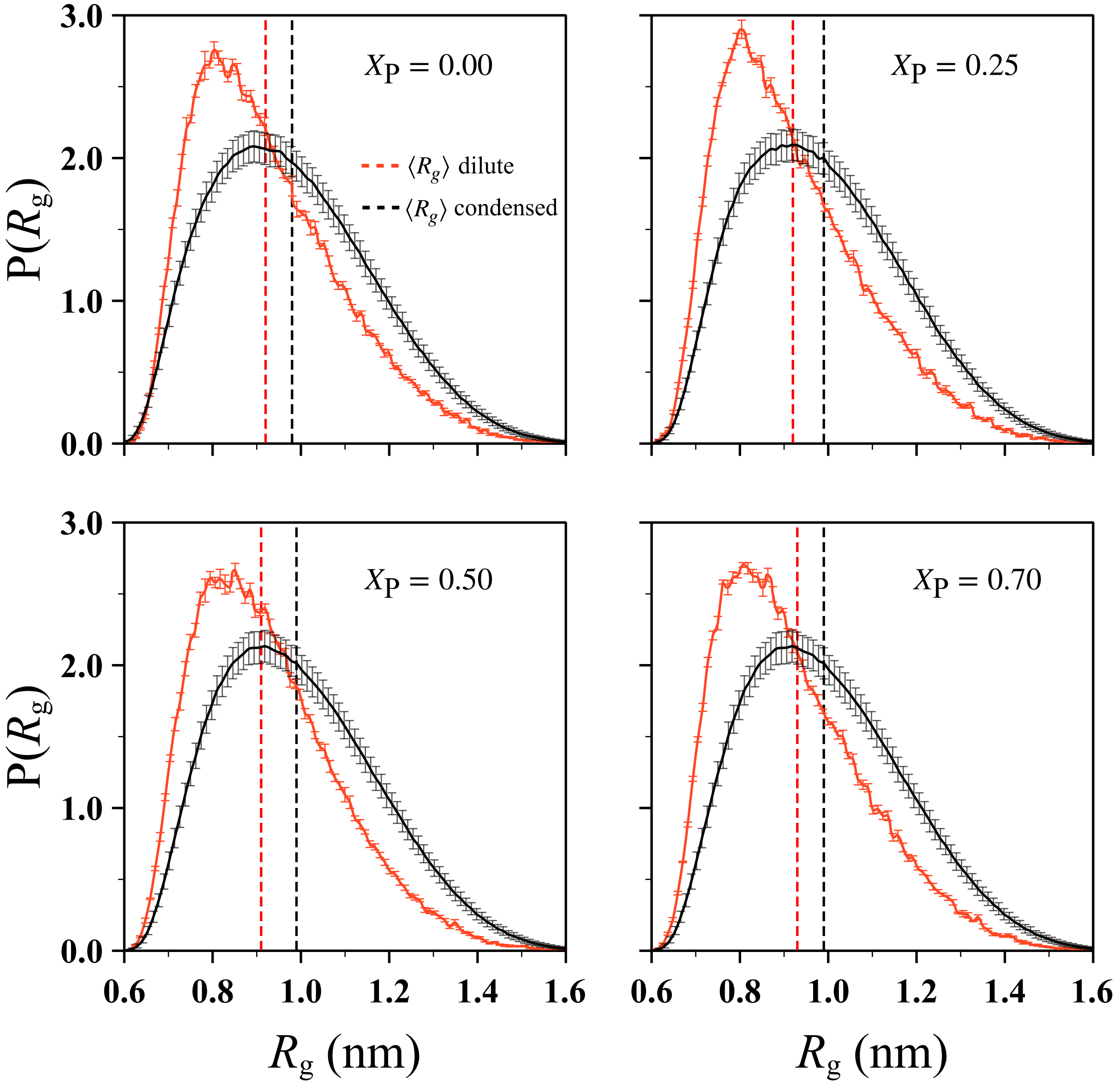

Figure S9: Probability distribution of radius of gyration R_g for the dilute phase (red) and condensed phase (black) for the HP+ model. The condensed phase values are computed as an average over all the chains in the system while the error bars are estimated as the standard deviation of R_g values among all chains. The dashed lines indicate the mean of the distributions.

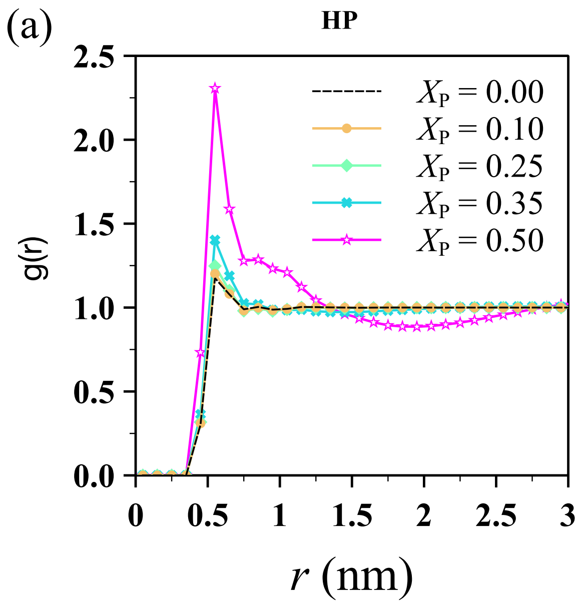

Figure S10: Radial distribution function $g(r)$ for different sequences that phase separated in the (a) HP model ($X_\mathrm{P} \leq X^*_\mathrm{P} = 0.45$) and (b) HP+ model ($X_\mathrm{P} \leq X^*_\mathrm{P} = 0.70$). The sequences that did not phase separate whose X_P is immediately above the threshold X^*_P (*i.e.*, $X_\mathrm{P} = 0.50$ in the HP model and $X_\mathrm{P} = 0.75$ in the HP+ model) are also shown for comparison.

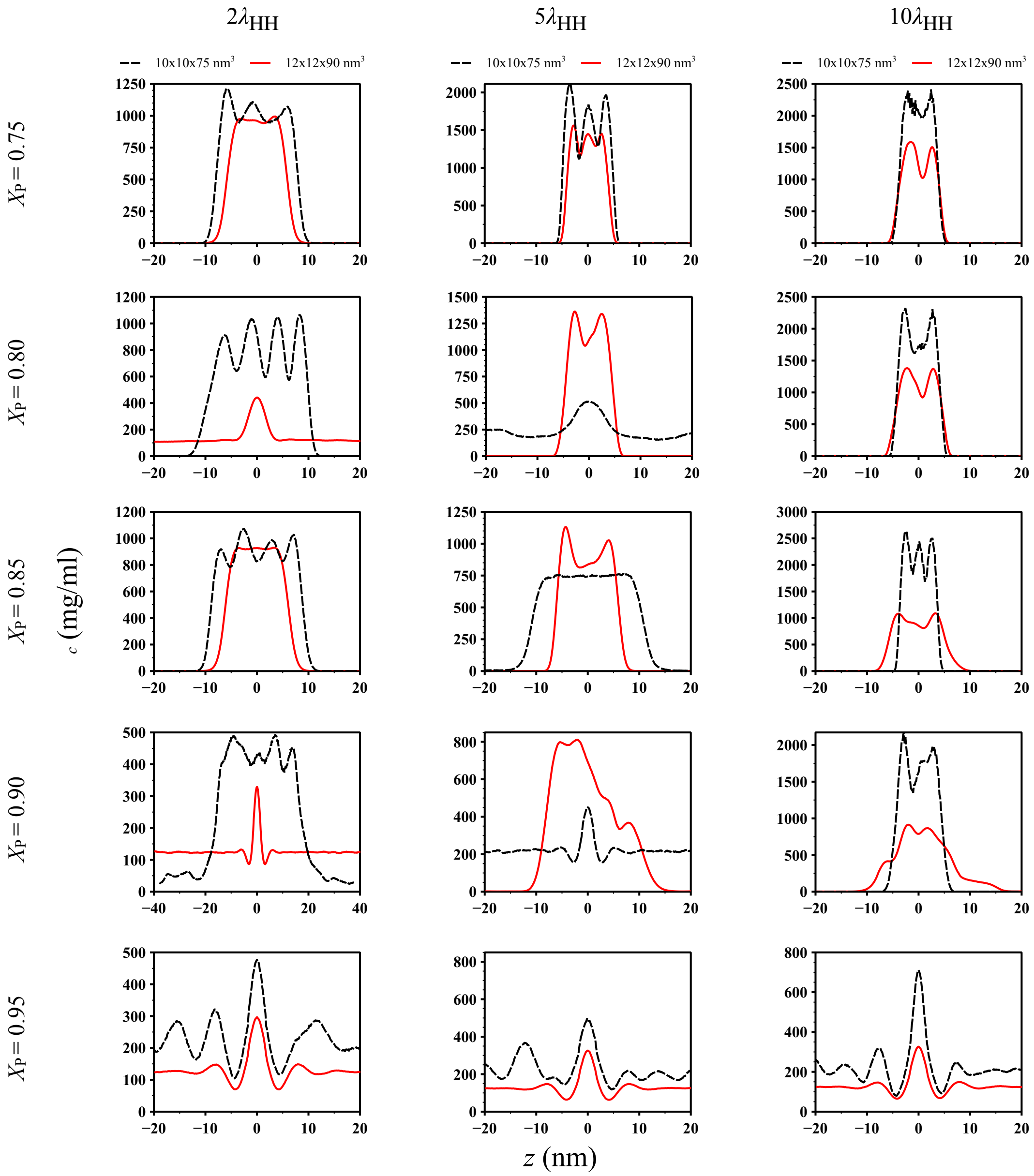

Figure S11: Concentration profiles for X_{P} values that did not undergo phase separation (*i.e.*, $X_{\mathrm{P}} > X_{\mathrm{P}}^{*} = 0.70$) in the HP+ model at $2, 5,$ and 10 times the interaction strength λ_{H} required to match the radius of gyration R_{g} to that of the purely hydrophobic chain in a rectangular box of two different sizes: $10\,\mathrm{nm} \times 10\,\mathrm{nm} \times 75\,\mathrm{nm}$ (dashed black lines) and $12\,\mathrm{nm} \times 12\,\mathrm{nm} \times 90\,\mathrm{nm}$ (solid red lines).

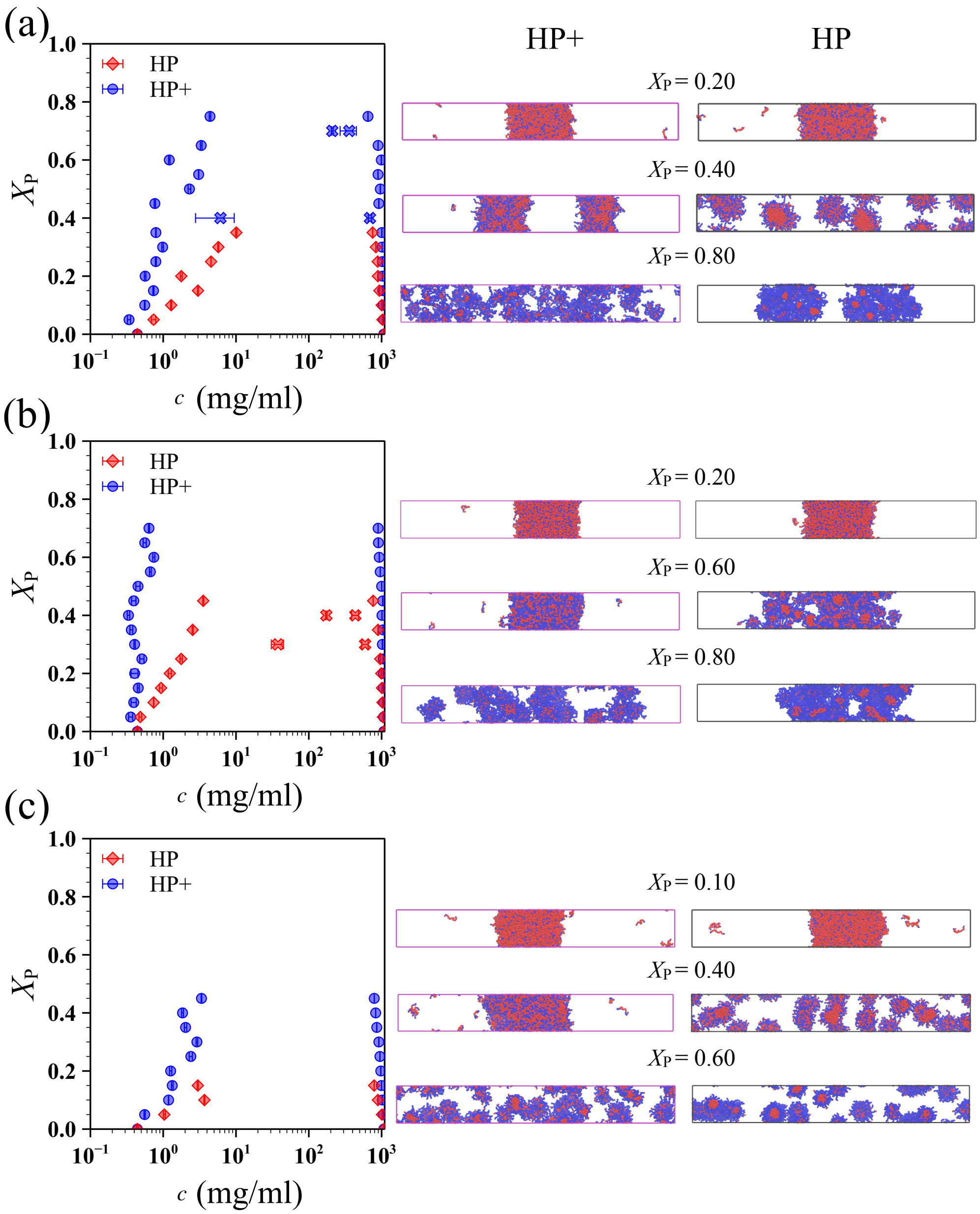

Figure S12: Phase diagrams and representative simulation snapshots for select X_P values in the HP and HP+ models: (a) RS1, (b) RS2, and (c) RS3. Data points shown as crosses for RS2 correspond to X_P values that did not phase separate even though they fell below the identified threshold X_P^* value.

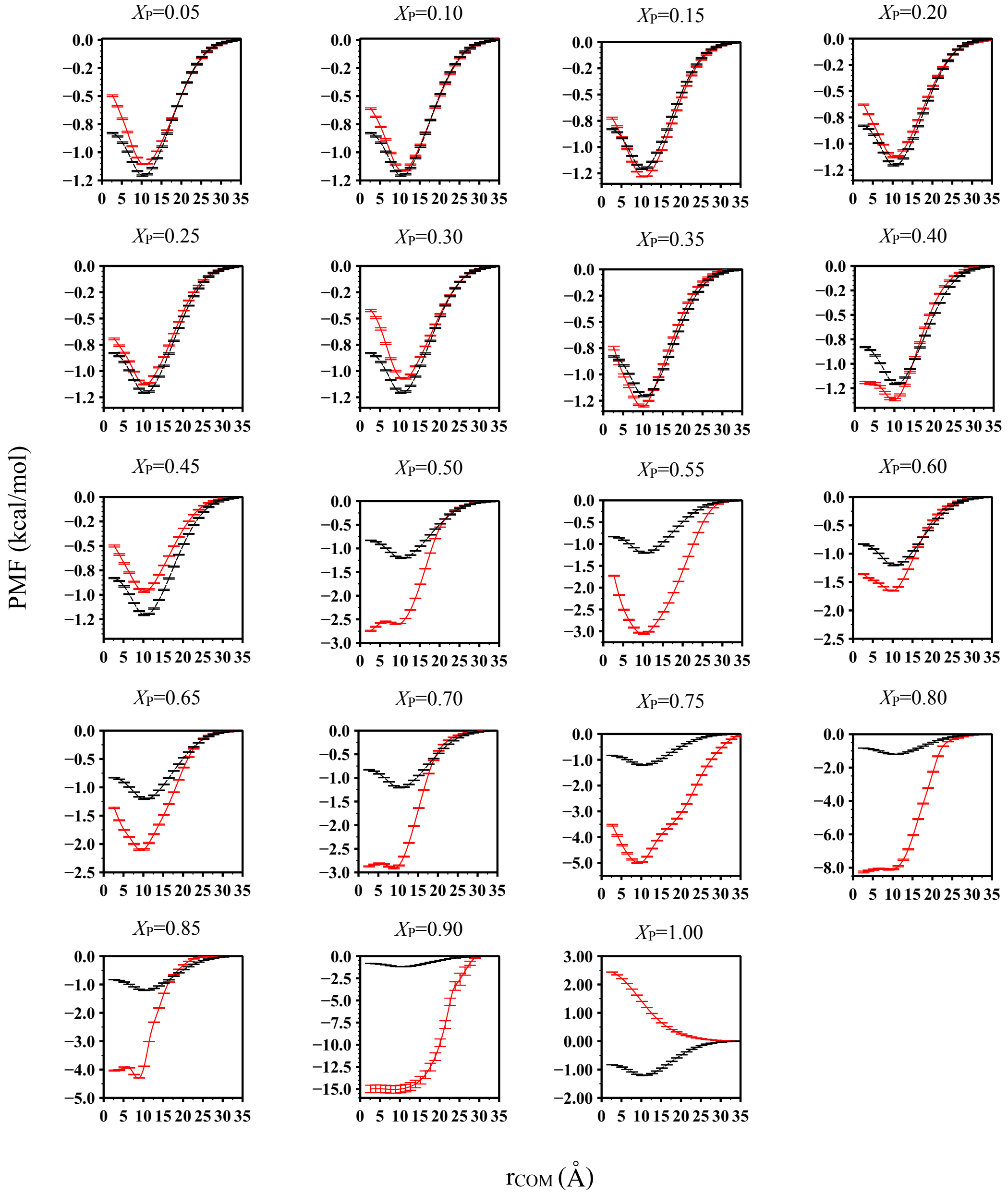

Figure S13: Potential of mean force (PMF) for all X_P values in the HP model. The PMF of the purely hydrophobic sequence ($X_P = 0$; black dashed line) is shown as a reference in all subplots. Scaling factor a to match the radius of gyration R_g for $X_P = 0.95$ cannot be obtained and hence, its PMF is not shown in the plot.

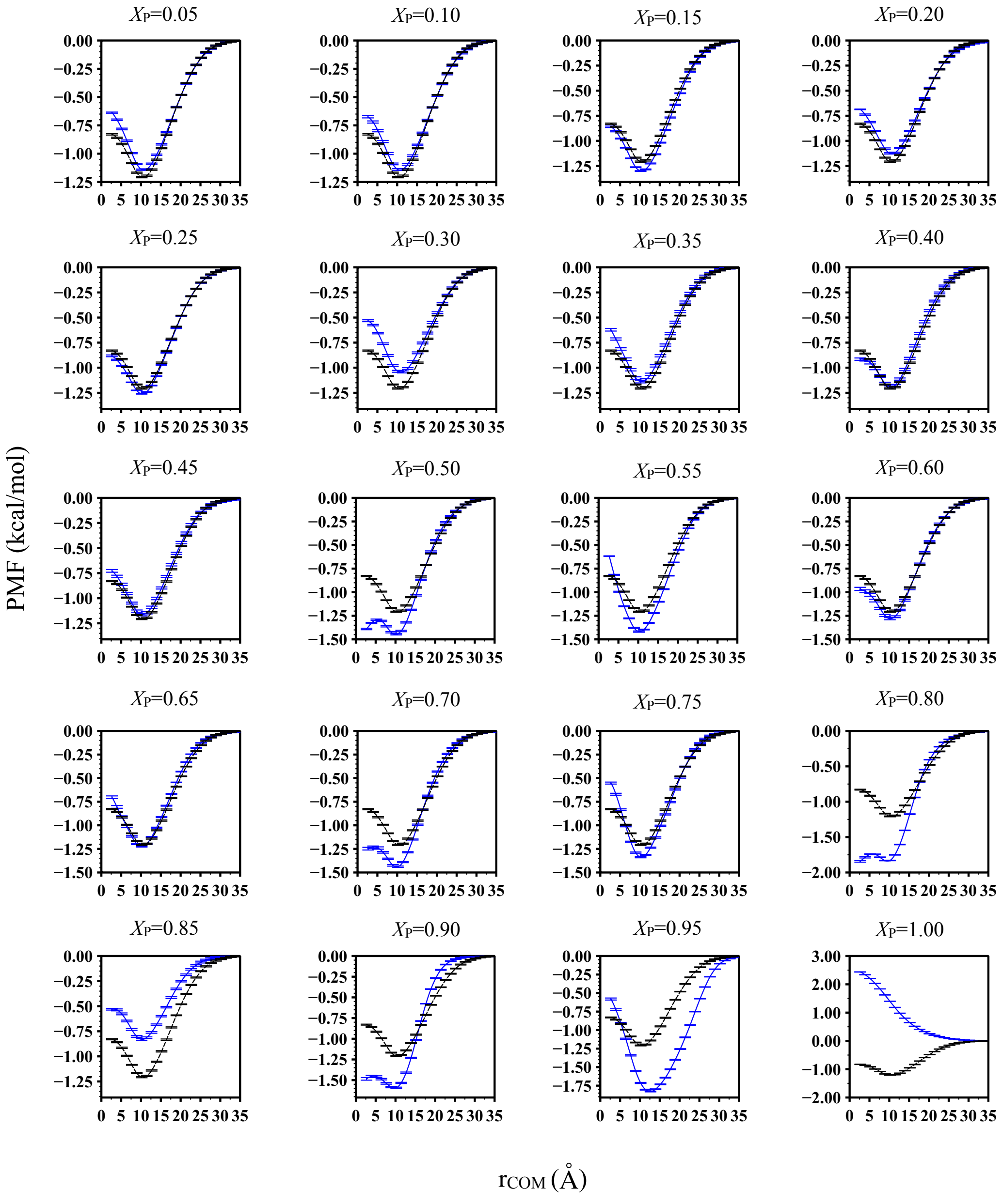

Figure S14: Potential of mean force (PMF) for all X_{P} values in the HP+ model. The PMF of the purely hydrophobic sequence ($X_{\mathrm{P}} = 0$; black dashed line) is shown as a reference in all subplots.

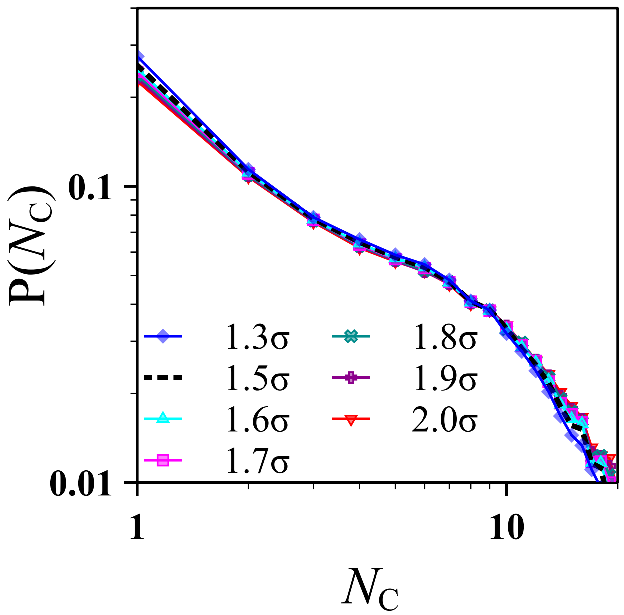

Figure S15: Probability of finding a chain in a cluster of size N_c for $X_P = 0.75$ sequence in the HP+ model. Cluster sizes were determined based on different cutoff distances as denoted in the legend, with $\sigma = 0.5\,\text{nm}$ being the monomer diameter. Note that the cutoff distance 1.5σ, shown as a dashed black line, was used to perform clustering analysis for different X_P values of the primary sequence set in Fig. 7.

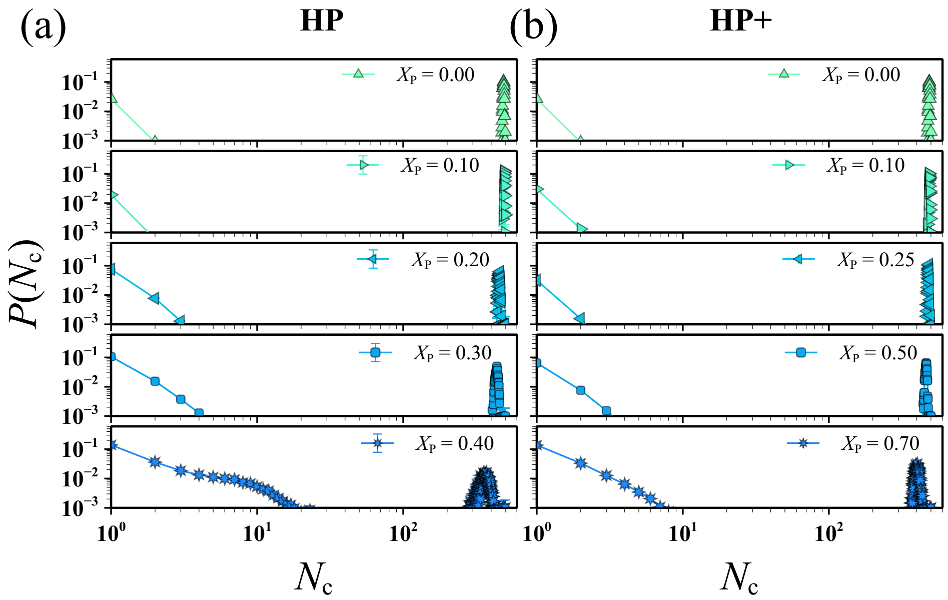

Figure S16: Probability of finding a chain in a cluster of size N_c for select X_P values that phase separated in the HP and HP+ models.

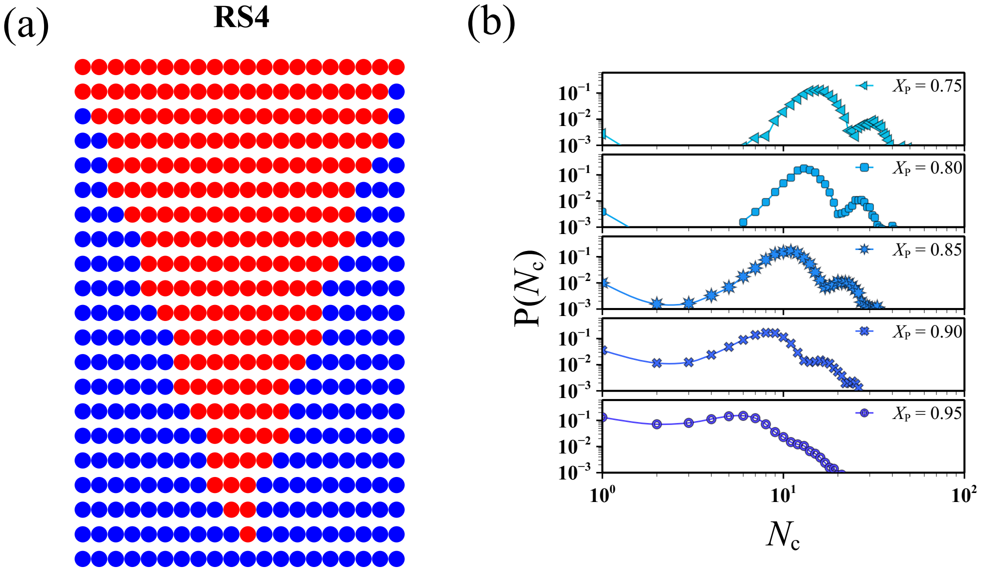

Figure S17: (a) Highly patterned sequence set (RS4) used to elucidate the effect of patterning on the formation of finite-sized aggregates. (b) Probability of finding a chain in a cluster of size N_c for select X_P values from RS4 that did not phase separate in the HP+ model.
